# Synergy of Rapamycin and Cyanoacrylate in Reducing Intimal Hyperplasia

**DOI:** 10.5935/abc.20180269

**Published:** 2019-01

**Authors:** Marcia Kiyomi Koike

**Affiliations:** Universidade de São Paulo, São Paulo, SP - Brazil

**Keywords:** Coronary Artery Diseases, Myocardial Revascularization, Vascular Diseases, Imunosupressive Agents, Cianoacrilates, Hyperplasia, Rats

Myocardial revascularization (CABG) surgery remains one of the main therapies for coronary disease. However, vascular disease that follows CABG remains a challenge to medicine. The mechanical, molecular and cellular changes undergone by the venous vascular graft when inserted into the coronary artery flow culminate in thrombosis, intimal hyperplasia, and atherosclerotic process, and end with restenosis.^1^^,^^2^

CABG experimental models were crucial to the understanding of mechanisms present in the course of vascular disease. The vascular graft model with anastomosis of the external jugular vein with the carotid artery represents an experimental model of vascular disease similar to that occurring after CABG. Pigs, rabbits, and rats may be used in this model because of protocol reproducibility, cost, and benefit.^3^^,^^4^

Strategies interfering with the mechanisms of vascular disease after CABG may reduce venous graft restenosis. Rapamycin, an immunosuppressive, showed antiproliferative effect of vascular smooth muscle cells in experimental models.^4^^,^^5^ Tianshu-Chu et al.,^6^ in an experimental study in rats that reproduces vascular disease after CABG, demonstrated that the combination of rapamycin and cyanoacrylate showed synergy to prevent intimal thickening. The combination of rapamycin and cyanoacrylate inhibited cell proliferation, primarily of dedifferentiated vascular smooth muscle cells (myofibroblasts) and vascular cells, preventing intimal hyperplasia, extracellular matrix deposition and neoangiogenesis. Thus, the combination of rapamycin with cyanoacrylate shows synergy when compared to the isolated use.
